# Mechanisms of action of bile acids in pediatric gastroesophageal reflux: Exploration through non-targeted and targeted metabolomics methods

**DOI:** 10.1371/journal.pone.0343229

**Published:** 2026-03-13

**Authors:** Linqian Zhang, Kang Wang, Qianwen Shi, Panjian Lai, Haiying Fang, Yuhe Chen, Xiaobing Li

**Affiliations:** Jinhua’s Key Laboratory of Birth Defects Prevention and Treatment, Jinhua Maternal and Child Health Care Hospital, Jinhua, Zhejiang, China; University of Milan, ITALY

## Abstract

**Purpose:**

To investigate the pathogenesis of pediatric bile reflux gastritis (BRG) using non-targeted and targeted metabolomics analyses of the changes and content differences of bile acids (BAs) in gastric juice.

**Methods:**

Data from 25 pediatric BRG patients treated at Jinhua Maternal and Child Health Care Hospital between May 2022 and August 2023 were retrospectively analyzed. Twenty-five patients with gastritis without bile reflux and 25 healthy controls were selected for the comparison of total bile acids (TBA) levels of gastric juice and peripheral blood and the identification of differential metabolites. The correlations of gastric juice and peripheral blood TBA levels with the clinical characteristics of the pediatric BRG patients were analyzed. ROC curves were constructed to evaluate the diagnostic performance of TBA in identifying BRG, as indicated by the area under the curve (AUC), sensitivity, and specificity.

**Results:**

Identification and quantitative metabolomics Studies have identified the top 10 differential metabolites in gastric juice between the BRG and healthy control groups, which include fatty acids, phospholipids, 23-carbon compounds, amines, vitamins, steroids, peptide hormones, monosaccharides, polyketides, and non-ribosomal peptides. This indicated that glycerophospholipid metabolism, prodigiosin biosynthesis, lysine degradation, glycerolipid metabolism, and primary BA biosynthesis were the most relevant pathways. Differential metabolites were primarily concentrated in the T helper 17 (Th17) cell differentiation pathway (P < 0.05, effect value>0.1).

**Conclusion:**

The metabolites and metabolic pathways identified in different groups will aid in elucidating the pathogenic mechanisms underlying pediatric BRG. Gastric juice and peripheral blood TBA levels showed a trend of aberrant expression in pediatric BRG patients, potentially guiding clinical diagnosis.

## Introduction

The molecular pathological mechanism of bile reflux gastritis (BRG) involves multiple factors and multi-link regulation imbalance, including lower esophageal sphincter (LES) dysfunction, decreased esophageal clearance ability, esophageal mucosal barrier damage, and prolonged acid exposure time, and shows complex changes at the molecular level such as protein expression, signaling pathway regulation, and gene polymorphism [1].

Traditional therapies have primarily focused on inhibiting gastric acid secretion. However, recent clinical observations have revealed that duodenal content reflux (bile acids, pancreatic enzymes) plays a key role in the pathogenesis of GERD, particularly exerting an important influence on the development of refractory GERD and its complications [2–4].Bile acids undergo conversion to non-ionic forms in acidic environments, leading to a surge in cytotoxicity that can dissolve the phospholipid layer of gastric mucosal epithelial cell membranes. After breaching the epithelial barrier, they activate pathways, resulting in abnormal contraction function of esophageal smooth muscle and stimulating the release of inflammatory mediators, triggering an inflammatory cascade. This process further activates intracellular signaling pathways such as MAPK and NF-κB, leading to downregulation, and paracellular permeability of epithelial cells increases, allowing H⁺and other harmful substances to more easily penetrate into the deeper layers of the mucosa, exacerbating cellular damage and inflammatory responses [5].

The treatment strategy for bile reflux has changed from simple acid inhibition to multi-target comprehensive intervention, among which bile acid targeted therapy has become the focus of clinical research. Based on the pathological role of bile acids in GERD, current medical treatment focuses on three core steps: neutralizing bile, improving bile composition, and promoting emptying, such as using bile acid binders (hydrotalcite) and bile acid composition regulators (ursodeoxycholic acid) [6,7].Compared with traditional acid-suppressive therapy, bile acids and their metabolites directly act on the damage factors, which not only relieve symptoms, but also interfere with the key pathological processes of the disease. In-depth research on the bile acid metabolism pathway also points the way for the development of new target drugs, and the interaction between intestinal flora and bile acid metabolism also provides a new perspective for treatment [8].Studies have confirmed that deoxycholic acid can promote the growth of intestinal polyps and accelerate the proliferation of tumor cells in mice. The mechanism may be related to the carcinogenic factors produced by intestinal flora metabolizing bile acids, suggesting that the regulation of intestinal flora and its metabolites may be a new way to prevent bile acid toxicity [9,10].

Although the gut microbiota has been extensively studied, metabolomics data, particularly metabonomics changes and differences in bile acids (BAs) within the gastric juice of patients with bile reflux gastritis (BRG), remain insufficient. The objective of this study is to construct a specific interaction network between BAs and metabolites in the gastric juice of children with BRG, and to investigate the impact of gastric juice BAs on clinical symptoms as well as the mechanism of immune injury mediated by gastric juice BAs, thereby facilitating future clinical applications.

## Materials and methods

### Experimental design and methods

The primary objective of this research was to examine alterations in the bile acid profile of patients with bile acid reflux gastritis (BRG) and to assess the impact of refluxed bile acids and the metabolome on the development of BRG. The collection and use of patient samples were approved by the ethics committee of Jinhua Maternal and Child Health Care Hospital (Ethics approval number: 2022KY009 2022/01/25). All included patients(between 2022/05/01 and 2023/08/31)were provided with a complete explanation of the nature of the study and provided written informed consent before undergoing endoscopic examination. Age- and sex-matched patients with a diagnosis of BRG (n = 25, Group A), patients with a diagnosis of gastritis without bile reflux ((BR-)G) (n = 25, Group B), and physically examined healthy controls without gastritis (n = 25, Group C) were recruited.

All patients underwent routine esophagogastroduodenoscopy (EGD) at our hospital. Patients undergoing treatment for *Helicobacterpylori* infection, using proton pump inhibitors or had previously undergone gastric and hepatobiliary surgery were excluded from the study.

A total of 75 patients who underwent EGD examination were included in this study. Endoscopy was performed early in the morning on patients who had abstained from food, water, and medications since the previous evening. The maximum possible amount of gastric mucus was aspirated prior to endoscopic examination. Gastric juice was aspirated into a collection container through the forceps channel of the endoscope.

According to the diagnostic criteria for BRG described in the Zhu Futang Practice of Pediatrics, the possibility of gastric reflux was considered when bile reflux (BR) was observed and yellow staining was present in the mucus pool and gastric mucosa after the gastroscope had been kept stationary for 1 min. A total of 10–20 mL of gastric juice was collected under fasting conditions to measure BA concentration in the gastric contents. A diagnosis of BR was made when BA concentration > 1 mmol/L or pH > 4.

### LC-MS/MS-based metabolomics data analysis

The LC-MS analytical platform was employed for non-targeted metabolomics analysis of gastric acid samples. The samples were first pre-treated for the removal of proteins and impurities. Subsequently, the metabolites were extracted, The mass spectrometric data were collected using UHPLC-Q Exactive HF-X Mass Spectrometer (Thermo Fisher, USA)) equipped with an electrospray ionization (ESI) source operating in positive mode and negative mode. Annotation of the metabolites and data pre-processing were performed using Progenesis QI software (Waters Corporation, Milford, USA) to obtain the metabolite list and data matrix. Differential metabolites were screened by combining the *t*-test and variable importance in projection (VIP) in the orthogonal partial least squares discriminant analysis (OPLS-DA) model. Biological information of the differential metabolites was mined using advanced analysis approaches, such as pathway analysis, correlation analysis, and cluster analysis.

### Targeted metabolomics analysis of gastric juice BAs

The LC-MS/MS analysis of sample was conducted on a ExionLC AD system coupled with a QTRAP® 6500 + mass spectrometer (Sciex, USA).The raw data were imported into Sciex software OS. All ion fragments were automatically identificated and integrated by using default parameters, besides, all intergration was checked manually. The metabolite concerntration of sample was calculated according to linear regression standard curve. Statistical analysis and graph generation were performed in SPSS 26.0 (IBM SPSS, USA) and GraphPad Prism 8.0 (GraphPad Software, San Diego, USA). Sample distribution was determined using the Kolmogorov-Smirnov test for normality. Normally and non-normally distributed data were statistically compared using one-way analysis of variance (ANOVA) and the Kruskal-Wallis test, respectively, and post-hoc comparison was conducted using Dunnett’s test. Relationships between BAs and pH were determined by calculating Spearman’s rank correlation coefficient. All P-values were adjusted to control the false discovery rate (FDR) using the Benjamini-Hochberg method. An adjusted P-value of <0.05 was considered statistically significant. LEfSe was performed to detect taxa with differential abundances across groups.

### Statistics

The data were processed using SPSS 26.0. The normally distributed metrological data were analyzed using Student’s *t*-test and presented as means ± standard deviations (SDs), whereas non-normally distributed metrological data were analyzed using the Mann–Whitney *U* non-parametric test. Two-sided P-values of < 0.05 were assumed to be statistically significant.

## Results

### Non-targeted metabolomics analysis of gastric juice

#### Results of raw data processing and analysis.

**Fig 1:**
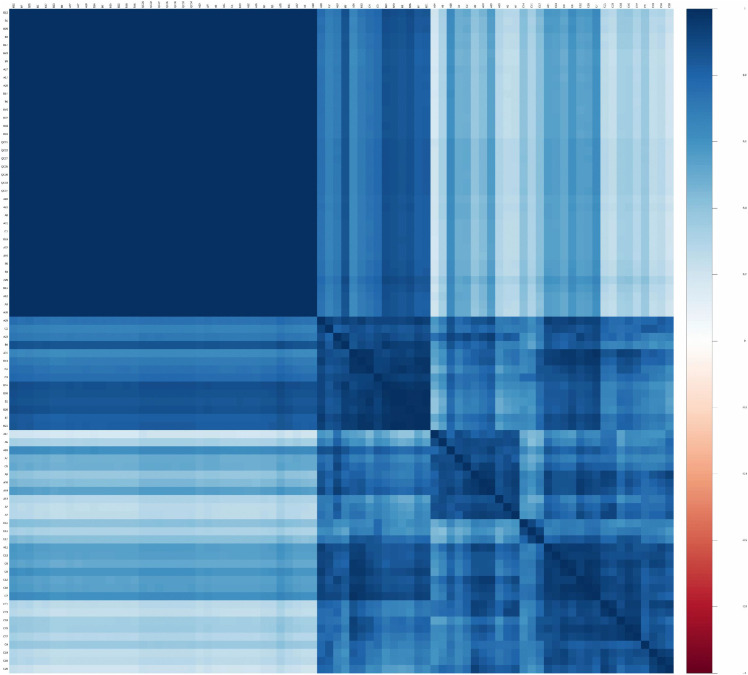
Heat map of correlation among the three groups of gastric juice samples. Sample names are plotted on the top and leftaxes. Each cell indicates the correlation between two samples, with different colors representing the relative magnitude of the inter-sample correlation coefficient. Branch lengths in the dendrogram indicate the relative distances between samples, with samples on the same branch being more similar.

**Intergroup correlation analysis:** Correlation analysis of the samples was performed using R language, and a sample correlation heatmap (Fig 1) was generated. A correlation coefficient close to 1 indicated high similarity in metabolite expression level between the samples. Data analysis was performed using principal component analysis (PCA). The PCA score plot showed a high degree of clustering of quality control (QC) samples, indicating good QC reproducibility and a stable analytical system.

### Differential analysis of sample metabolomics data

#### Multivariate analysis (OPLS-DA).

**Fig 2:**
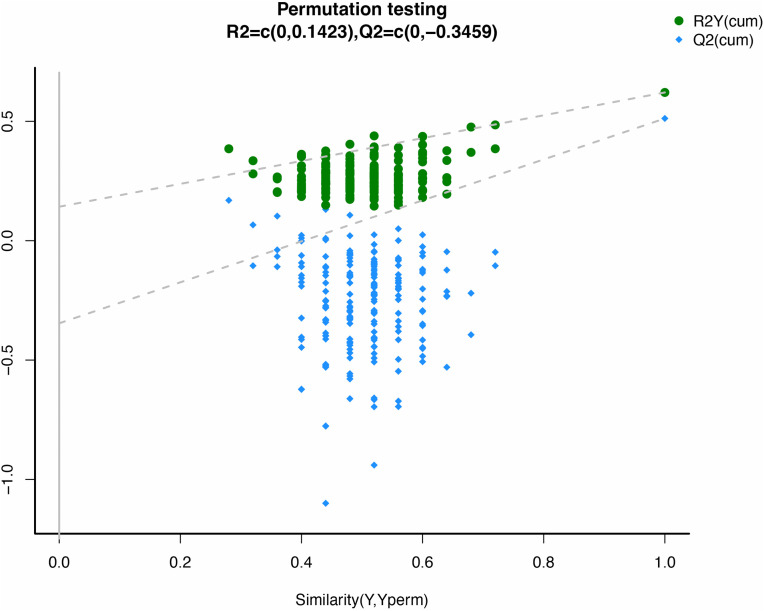
Differences in metabolic profile between BRG group and Control group.

For a more accurate representation of metabolic differences among the three groups, a supervised OPLS-DA model was adopted. The results of permutation testing for the OPLS-DA model used in the study are illustrated in the permutation test plot (Fig 2). The original R2 points at the far right end remained higher than the R2 points at the left end, and the original Q2 points at the right end remained higher than the Q2 points at the left end. The OPLS-DA model demonstrated no signs of overfitting, indicating its stability and effectiveness in explaining the differences between the two sample groups.

### Screening and analysis of differential metabolites

**Fig 3:**
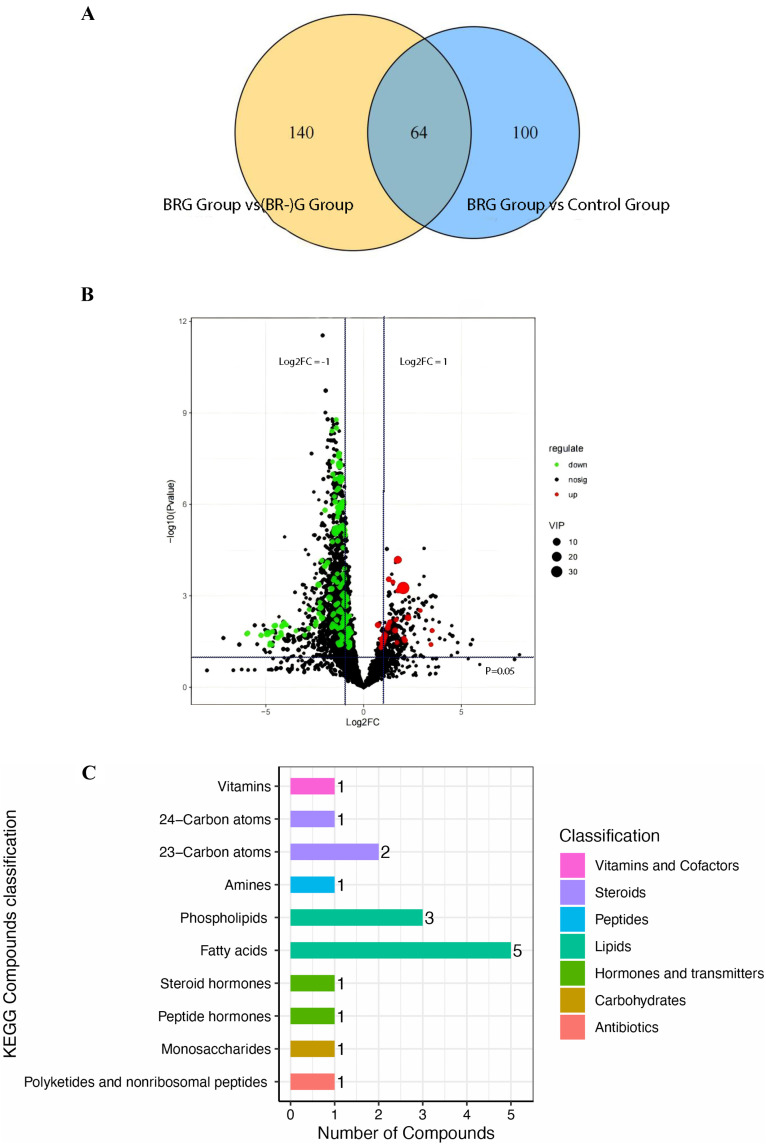
Analysis of differential metabolites. **(A)** Venn diagram of differential metabolites between BRG group and others. **(B)** The Volcano Plot between BRG Group and Control Group.

UPLC-MS/MS was employed for metabolomics analysis of gastric juice. A total of 3,707 metabolites were detected from the three groups of samples. Fig 3A shows the comparative analysis of differential metabolites among the three groups (VIP > 1 and P < 0.05). Volcano plots, generated based on fold changes in differential metabolite expression between the two groups, revealed 164 differential metabolites between the BRG and Control groups, with more upregulated metabolites in the BRG group than in the Control group(Fig 3B). Differential metabolites were categorized based on structure and function. Fig 3C displays the top 10 differential metabolite categories, with the fatty acid category having the highest number significant difference.

The vertical axis represents the statistical test values (log10(p_value)) for differences in metabolite expression, where higher values indicate more significant differences. Both the horizontal and vertical axes display log-transformed values. Each dot represents a specific metabolite, and the dot size indicates the VIP value. Dots on the left represent differentially downregulated metabolites, and dots on the right represent differentially upregulated metabolites, with dots further to the left, right, and top having more significant differences in expression. **C. Bargraph of differential metabolite classification.** The horizontal axis represents the number of metabolites, and the vertical axis represents the metabolite category.

### Cluster analysis of differential metabolites

**Fig 4:**
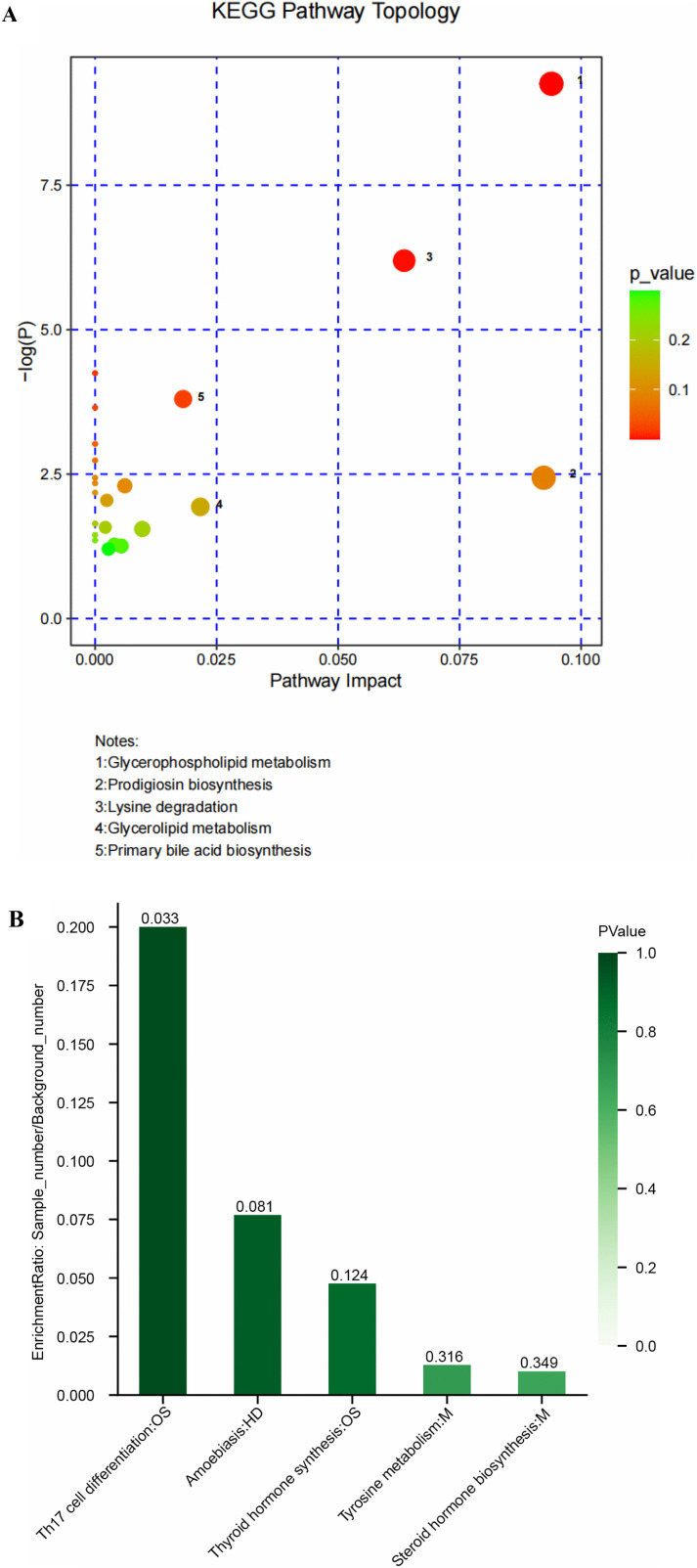
Cluster analysis of differential metabolites. **A. Bubble plots of KEGG topological analysis (BRG group and Control group).** Each bubble in the chart denotes a KEGG pathway. The horizontal axis represents the magnitude of the impact value of metabolites in each pathway, and the vertical axis represents the significance of metabolite involvement in pathway enrichment (-log2(P-value)). Bubble size indicates the magnitude of the impact value, with larger bubbles having a greater impact. Different colors denote different P-values of pathway enrichment. **B. Column chart of KEGG pathway enrichment analysis of differential metabolites (BRG group and Control group). Each column in the chart represents a specific pathway, with the height indicating the level of enrichment.** pathway. The text on the horizontal axis indicates the name and category of the pathway, and the description of the category is shown in the upper left corner. The height of the column represents the enrichment coefficient, which is calculated by the ratio of the number of samples to the background number. Column color indicates the significance of enrichment (i.e., P-value), with darker colors indicating more significant pathway enrichment. A darker shade corresponds to a lower P-value, with ***P < 0.001 indicating very high significance, **P < 0.01 indicating high significance, and *P < 0.05 indicating moderate significance. The color gradient on the right shows the colors corresponding to the various P-values.

After matching the information of differential metabolites screened and identified through the process described above, enrichment and topological analyses were performed using the pathway databases of the corresponding species. The bubble chart of KEGG topology analysis results indicated that the pathways most significantly impacted by differential metabolites between groups A and C were, in descending order of impact, glycerophospholipid metabolism, prodigiosin biosynthesis, lysine degradation, glycerolipid metabolism, and primary BA biosynthesis (Fig 4A). The KEGG pathway enrichment analysis of differential metabolites across various groups revealed that the metabolites differentiating groups A and C were predominantly associated with the T helper 17 (Th17) cell differentiation pathway, as indicated by a statistically significant P-value 0.032 (< 0.05) and a substantial effect size (> 0.1). (Fig 4B).

### BA-targeted metabolomics analysis of gastric juice

#### Clinical characteristics of pediatric patients.

**Fig 5:**
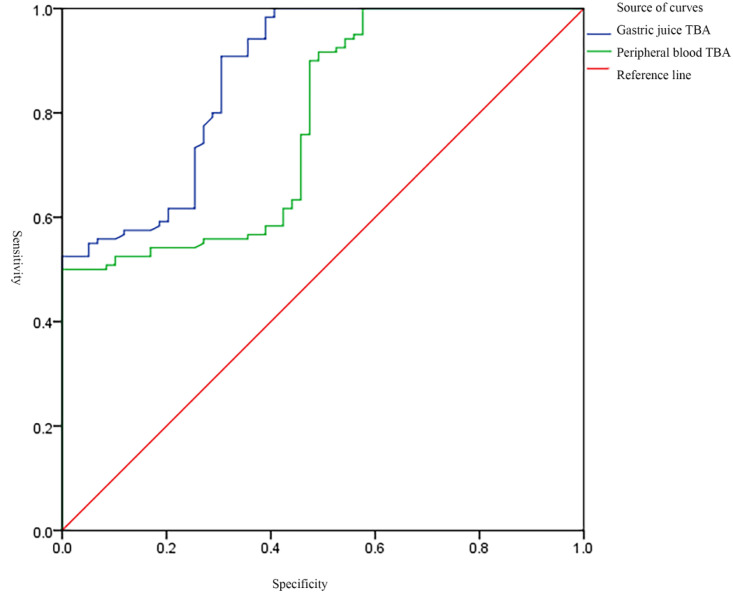
Performance analysis of gastric juice and peripheral blood TBA levels in the diagnosis of BRG.

The clinical data of the three groups, such as gender, age, PH of gastric juice, TBA of gastric juice and peripheral blood, were statistically analyzed, the gastric juice pH was significantly higher in BRG patients than in both gastritis controls without bile reflux and healthy controls, confirming the alkaline shift due to duodenal content reflux (Table 1). The receiver operating characteristic (ROC) curve was drawn to analyze the efficacy of TBA of gastric juice and peripheral blood in the diagnosis of BRG, and P < 0.05 was considered statistically significant. ROC analysis showed that the AUC values of TBA in gastric juice and peripheral blood for the diagnosis of BRG were 0.872 and 0.780, respectively (P < 0.05). The sensitivity were 90.80% and 50.00%; The specificity were 69.50% and 96.60%, respectively. (Fig 5).

**Table 1:** Clinical characteristics of healthy controls (C), BRG patients (A), and (BRG-)patients (B).

	A (N = 25)	B (N = 25)	C (N = 25)
**Sex (male/female)**	12/13	10/15	16/9
**Age (years)**	10.2 ± 2.9	8.7 ± 3.2	5.3 ± 3.4
**pH**	5.76 ± 0.43	2.17 ± 0.71	1.68 ± 0.82*
**Gastric juice TBA (ng/L)**	13.55 ± 4.74	9.5 ± 2.08	8.08 ± 1.83***
**Serum TBA (umol/L)**	11.24 ± 3.04	8.1 ± 2.61	7.71 ± 2.05***

*P < 0.05, **P < 0.01, ***P < 0.001

### Conjugated BA levels were highly correlated with BRG

**Fig 6:**
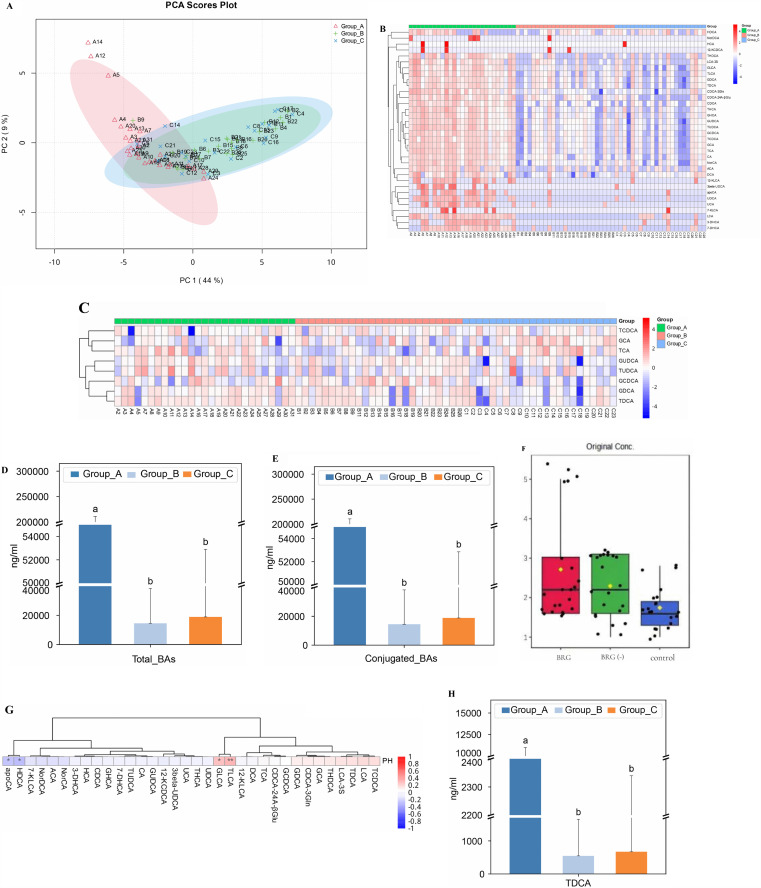
Multivariate analysis of BA-targeted metabolomics data from gastric juice samples. **A. PCA score plot. B. Sample correlation heat map. C. Heat map of normalized concentrations of conjugated BAs in gastric juice.** The color of each cell in the heat map corresponds to the normalized and log-transformed original abundance of each BA in each sample. **D. Concentration of total BAs in gastric juice. E. Concentration of conjugated BAs in gastric juice. F. pH of gastric juice. G. Heat map of Spearman’s correlation coefficients of two elevated BAs (TLCA, GLCA), 2 decreased BAs (HDCA, apoCA), and pH in gastric juice.** Cell colors indicate the values of the Spearman correlation coefficient **(r)**. **P < 0.01, *P < 0.05. **H. Concentration of TDCA in gastric juice (mean).** Differences between groups were assessed using the Kruskal-Wallis test. **: P < 0.01, *: P < 0.05.

To investigate the BA characteristics of BRG, we utilized LC-MS/MS and GC-MS/MS techniques for the multivariate analysis of BA-targeted metabolomics data from gastric juice samples collected from control (n = 25), BRG (n = 25), and (BR-)G (n = 25) groups.EGD revealed a large amount of yellow-green fluid in the gastric lumen of BGG patients. The BA profiles of gastric juice samples of the three groups were measured using a targeted metabolomics approach. PCA indicated that Group A significantly differed from Groups B and C, while Groups B and C largely overlapped (Figs 6A–B). The TBA (sum of all detected conjugated and unconjugated (free) BAs) level of BRG patients was increased compared with the healthy controls (Group C). In particular, conjugated BA levels were significantly elevated (Figs 6D–E), with glycocholic acid (GCA), glycochenodeoxycholic acid (GCDCA), glycodeoxycholic acid (GDCA), glycoursodeoxycholic acid (GUDCA), taurocholic acid (TCA), taurochenodeoxycholic acid (TCDCA), taurodeoxycholic acid (TDCA), and tauroursodeoxycholic acid (TUDCA) showing significant increases. Among the various BAs, TUDCA exhibited the greatest difference (VIP = 1.56, fold change (FC)=39.1) (Fig 6C).

The rise in BAs raised the question of whether disturbances had occurred in the natural acid-base balance stomach. To address this question, we measured the pH of gastric juice samples of the three groups. The results indicated that the pH was within the normal range for gastric acid, which typically falls between 0.9 and 1.8.The levels are considerably higher in patients with BRG (5.76 ± 0.43) and (BR-)G (2.17 ± 0.71) compared to healthy controls (1.68 ± 0.82) (Fig 6F).In contrast to strong stomach acid, BAs that reflux from the duodenum to the stomach are weak acids, which can disrupt the natural acid-base balance and elevate the intragastric pH.We identified strong positive correlations between the levels of conjugated BAs and the pH of human gastric juice, particularly involving taurolithocholic acid (TLCA) and glycolithocholic acid (GLCA), showing the strongest positive correlations with pH (Fig 6G). The TDCA level in gastric juice was significantly elevated in the BRG group (Fig 6H).

BRG: bile reflux gastritis; GCA: glycocholic acid; GCDCA: glycochenodeoxycholic acid; GDCA: glycodeoxycholic acid; GUDCA: glycoursodeoxycholic acid; TCA: taurocholic acid; TCDCA: taurochenodeoxycholic acid; TDCA: taurodeoxycholic acid; TUDCA: tauroursodeoxycholic acid; BAs: bile acids.

## Discussion

Bile reflux (BR), characterized by the retrograde flow of duodenal contents such as bile, pancreatic juice, and duodenal juice into the stomach, has been identified as an independent risk factor for chronic atrophic gastritis (CAG). Extensive epidemiological studies have substantiated this association, indicating that BR significantly contributes to the development of CAG, intestinal metaplasia (IM) and gastric cancer (GC) [5,11]. Our study also confirmed that gastric juice PH was higher in patients with bile reflux than in healthy children and those without bile reflux. The reflux of alkaline bile into the stomach can considerably alter the gastric environment and shape the composition of the gastric microbiota, then this change are closely related to the development of GC and gastric mucosal lesions [12].

BAs are not merely digestive surfactants but also serve as important cell-signaling molecules that stimulate several signaling pathways to regulate several biological processes [13]. BAs exist in free and conjugated forms. In BR, the stomach has a higher pH and greater accumulation of conjugated BAs, which leads to ongoing damage of the gastric epithelial cells [14,15]. A significant increase in BAs, especially conjugated BAs, in the gastric juice of BRG patients elevates intragastric pH, ultimately increasing the amount of lipopolysaccharide (LPS)-producing bacteria in the stomach [16,17]. The abundance of LPS-producing bacteria is positively correlated with the level of TDCA in gastric juice. This is consistent with our findings, which indicated a significant increase in the TUDCA and TDCA levels of the gastric juice of pediatric BRG patients. Recent research has progressively uncovered the intricate role of bile acids in gastroesophageal reflux disease (GERD), establishing them as a novel therapeutic target for managing the condition. For example, FXR activation in hepatocytes has been shown to inhibit bile acid synthesis and accumulation and induce the expression of bile acid efflux pumps, which are critical for attenuating bile acid-mediated mucosal injury [18]. Ursodeoxycholic acid (UDCA) can reduce the incidence of bile reflux and gastritis in patients with gastric cancer after gastrectomy by about 50% [6].Our findings also provide new ideas for the treatment of BRG in children. Targeted reduction of conjugated bile acids, especially TDCA and TUDCA, can alleviate gastric epithelial cell damage.

Microbiome-targeted therapy strategies have shown unique promise. Previous studies have also demonstrated that bile reflux significantly alters the distribution of bile acid in the oral cavity, stomach and intestine, and alters the gut microbiota [19].In a recent study, the functions of 207 potential hydroxysteroid dehydrogenase (HSDH) enzymes within the gut microbiota were systematically characterized, presenting a comprehensive ‘panorama’ of microbial bile acid synthesis was constructed, and 56 novel bile acids were identified. Certain bile acid molecules, produced by the gut microbiota, have been identified to have potent inhibitory effects on androgen receptor (AR) activity, as demonstrated in a study published in Cell. Recent studies have identified novel bile acids produced by gut microbiota, which have demonstrated significant anti-tumor activity in animal models. Specifically, the bile acid 3-oxo-Δ4,6-LCA has been found to inhibit tumor growth and enhance the effects of anti-PD-1 therapy, providing strong evidence for the immunomodulatory effects of bile acid modulators. It also provides new ideas for the prevention and treatment of GERD-related Barrett’s esophagus and tumors [20].

Our non-targeted metabolomics identified the Th17 cell differentiation pathway as the most significantly enriched among differential metabolites (P < 0.05, impact >0.1), alongside other altered pathways including glycerophospholipid metabolism, prodigiosin biosynthesis, and lysine degradation (Fig 4). This suggests a potential link between bile acid reflux, specific microbial or host metabolic shifts, and local gastric immune dysregulation. We hypothesize that the increased conjugated bile acids (e.g., TDCA, TUDCA) in the gastric lumen may, directly or indirectly through modulating the gastric microbiota, influence the local metabolic milieu. This altered metabolism could, in turn, favor a pro-inflammatory Th17 cell response, as indirectly indicated by the enrichment of its regulatory pathway in our data. This is consistent with previous findings of reflux esophagitis in adults, showing that patients with reflux esophagitis (RE), compared with healthy people,which indicate that patients with reflux esophagitis (RE) have an elevated T helper 17/regulatory T cell ratio (Th17/Treg) and increased expression of regulatory factors such as RORγt, interleukin (IL)-17, IL-6, and TGF-β,reduction of Foxp3,IL-10 levels compared to healthy individuals. In other words, Th17/Treg may be involved in the development of RE through the regulation of the release of inflammatory cytokines [21]. Th17 cells may considerably contribute to many of these processes as they are central to inducing inflammation, activating antimicrobial responses, and influencing cell infiltration in tumor environments [22].Several studies on pediatric patients infected with *H. pylori* have provided evidence that Treg cell responses were demonstrated a positive correlation with bacterial density, while Th17 responses exhibited a negative correlation with bacterial density [23]. In a study comparing *H. pylori*-infected adults and children, Serrano et al. reported that the children had higher numbers of IL-10 cells than adults. Consequently, the infected children showed diminished gastric Th17 responses, which were linked to a reduction in gastritis [24]. However, the significance of these findings in children with bile reflux gastritis still needs to be further verified by functional experiments.

Based on the structure and function of metabolites, the differential metabolites between Groups A and C were categorized. The top 10 differential metabolites were fatty acids, phospholipids, 23-carbon compounds, amines, vitamins, steroids, peptide hormones, monosaccharides, polyketides, and non-ribosomal peptides. Fatty acids exhibited the most significant difference (Fig 4C) and corresponded to the following pathways: glycerophospholipid metabolism, prodigiosin biosynthesis, lysine degradation, glycerolipid metabolism, and primary BA biosynthesis. Prodigiosin, a secondary metabolite known for its anticancer properties, is part of the prodiginine family and has been studied for its potential in treating various types of cancer. Produced by the Gram-negative bacteria *Serratia marcescens*, it possesses anticancer properties and potentially plays a role in modulating and to selectively suppress the proliferation and immune functions of T cells [25]. Lysine has been suggested as a non-specific bridging molecule that links antigens to T-cells, thereby allowing T-cells to produce specific effects against antigens. Lysine also stimulates the secretion of pepsin and gastric acid, thereby enhancing the efficiency of gastric juice secretion [26]. Notably, the co-enrichment of “prodigiosin biosynthesis”and “lysine degradation”involved in immune cell function—points to a previously underappreciated potential interplay between bile-altered gastric ecology, bacterial metabolites, and host mucosal immunity. The specific role of these pathways in pediatric BRG pathogenesis is a novel aspect revealed by this study and represents a new direction for mechanistic inquiry

Patients with chronic gastritis often suffer from anxiety, depression and other emotional disorders due to persistent abdominal pain, bloating, fatigue and other symptoms. This kind of psychological stress not only aggravates the patient’s pain, but also affects the digestive function through the “brain-gut axis” mechanism, forming a vicious circle of symptoms and negative emotions. Patients with bile acid-related GERD face unique psychological challenges. The symptoms, such as intractable epigastric burning pain and bilious vomiting resulting from bile reflux, do not respond well to traditional acid suppression treatment, which can easily give rise to a sense of ineffectiveness and despair. Especially after a prolonged period of ineffective treatment, patients often develop “treatment burnout”, harbor doubts about follow-up treatment recommendations, and this affects their treatment compliance. In addition, Studies have shown a correlation between bile reflux and an increased risk of developing Barrett’s esophagus and esophageal adenocarcinoma, which can lead to heightened cancer-related anxiety among patients and exacerbate their psychological distress. Therefore, personalized treatment for BRG is urgent.

The development of bile acid-targeted therapy faces multiple challenges in terms of accessibility, such as research and development, supply chain, and payment systems. The complexity of bile acid signaling pathway constitutes the primary technical bottleneck. Multidimensional solutions to These challenges are currently under exploration. Future research should focus on developing precision medicine strategies.Patient stratification based on biomarkers (such as bile acid composition, receptor expression patterns, and microbial metabolic characteristics) is the key to achieving individualized treatment.

## Conclusion

Pediatric BRG possesses specific BA metabolism characteristics. The elevation of conjugated BA levels correlates with pH, with TLCA and GLCA having the strongest correlations. These markers enable the prediction of BRG severity. A total of 63 differential metabolites were identified and analyzed from the gastric juice of children with gastroesophageal reflux using GC-MS-based non-targeted and targeted metabolomics techniques. Utilizing correlation analysis, the five most significant metabolic pathways were identified by examining differential metabolites and their associated pathways. Among the differential metabolites, one was involved in the lipid metabolism pathway, two in the the metabolism of cofactors and vitamins involves two processes in exogenous biodegradation and metabolism, and three in amino acid metabolism. These findings suggest two concrete, testable therapeutic avenues beyond acid suppression: (1) Targeted reduction of specific conjugated bile acids, particularly TDCA and TUDCA, which were most elevated and correlated with pH changes; and (2) Modulation of the gastric microbiota or its metabolic output to counteract the predicted pro-inflammatory Th17 bias and the potential influence of bacterial metabolites like those in the prodigiosin pathway. While broader challenges in drug development and patient access exist, these data-linked targets provide a focused starting point for preclinical intervention studies in pediatric BRG.

### Limition

This study has several limitations. The sample size, while sufficient for an initial metabolomics screen, is modest. The causal relationship between the identified metabolic shifts (e.g., in Th17 pathway metabolites, prodigiosin-related compounds) and BRG pathology remains associative. Future studies should: (1) Validate these metabolic signatures in a larger, independent pediatric cohort; (2) Employ functional models (e.g., bile acid-exposed gastric organoids or specific pathogen-free animal models) to test the direct effect of key conjugated BAs like TDCA on Th17 differentiation and the expression of prodigiosin biosynthesis genes; and (3) Perform integrated multi-omics analyses (metagenomics alongside metabolomics) on gastric juice samples to directly link specific bacterial taxa with the production of immunomodulatory metabolites like those in the prodigiosin pathway.
